# Comparison of the Effectiveness of Mindfulness-Based Stress Reduction and Compassion-Focused Treatment on the Severity of Gastrointestinal Symptoms in Patients with Irritable Bowel Syndrome

**DOI:** 10.34172/mejdd.2024.370

**Published:** 2024-01-31

**Authors:** Tahereh Pourkazem, Ahmad Ghazanfari, Reza Ahmadi

**Affiliations:** ^1^Department of Psychology, Faculty of Humanities, Shahrekord Branch, Shahrekord Islamic Azad University, Shahrekord, Iran

**Keywords:** Mindfulness-based stress reduction, Compassion-focused therapy, Gastrointestinal symptoms, Irritable bowel syndrome

## Abstract

**Background:**

The aim of this research was to compare the effectiveness of mindfulness-based stress reduction and compassion-focused on the severity of digestive symptoms in patients with irritable bowel syndrome (IBS).

**Methods:**

The research method was of semi-experimental type with pre-test, post-test, follow-up, and experimental and control groups. The population included patients with IBS in Isfahan city, 45 of them were selected by convenience sampling method and randomly assigned to three groups (15 in each group). Then, the patients of one experimental group received eight sessions of 90 minutes of a mindfulness-based stress reduction program, while the other experimental group received eight sessions of 90 minutes of compassion-focused therapy. The measurement tools included the severity of IBS scale and a short clinical interview. Research data were analyzed using variance analysis with repeated measures on one factor (mixed design).

**Results:**

The results showed that both methods of intervention had been equally effective on the severity of disease symptoms (*P*<0.01).

**Conclusion:**

Both intervention methods can be used as a complementary treatment for patients with IBS.

## Introduction

 Over the past 20 years, the definition of irritable bowel syndrome (IBS) has evolved based on studies that have identified symptoms that discriminate those labeled as IBS from organic disease, but historically, this disease has been known since 150 years ago. Currently, its description and diagnosis are based on the Rome IV diagnostic criteria that was developed in 2016.^1^ IBS is one of the most common digestive disorders, characterized by altered bowel habits in association with abdominal discomfort or pain in the absence of detectable structural and biochemical abnormalities.^2^

 IBS is the most common, costly, and debilitating disorder among the 20 digestive system functional disorders.^3^ The prevalence of this disorder in Iran is reported to be 11%-25% among adults, and is more common among women.

 IBS creates a major burden for the individual and society and causes significant financial and non-financial damages.^4^ The diagnostic criteria for IBS include abdominal pain or discomfort for 12 weeks with at least two of the following three characteristics: relief of pain with defecation, onset of symptoms with a change in frequency of defecation, and the onset of symptoms with a change in stool appearance.^5^

 Surprisingly, most patients who suffer from these intestinal disorders do not know that their emotional states are affected by their problems.^6^

 The etiology of IBS is complex and multifactorial, and reasons such as abnormal gastrointestinal movements, visceral hypersensitivity, and psychological factors have been confirmed in studies.^7^ The prevalence and severity of stress, anxiety, and depression are closely related to the onset and severity of symptoms in patients with IBS.^8^

 It is also associated with a high prevalence of mental disorders, and consequently, these patients experience a higher level of anxiety and depression than the control group.^9^

 Psychological disorders are often suggested as the main cause of this disease. According to the studies conducted, among patients with IBS, the prevalence of psychological stress is about 20-50%, and 50%-90% of patients will experience a psychological disorder during their life, such as anxiety disorders, especially generalized anxiety disorder, depression, and somatoform disorders.^10^

 Despite the relatively high prevalence of IBS, this disorder has not been well managed in the health system due to its unclear nature, which in turn has led to frustration and dissatisfaction among patients and doctors.

 Obviously, these problems can play a role in both diagnostic processes and poor treatment results.^11^

 In fact, since IBS is a functional disorder and does not have a specific physical or biological cause, it is classified as a psychosomatic disease, and given its close relationship with psychological symptoms, measuring psychological symptom levels among these patients is one of the best ways to evaluate the improvement of symptoms and the effect of treatment.^12^

 In addition, psychological stress is also considered one of the factors influencing immune system activity. Hence, more studies are needed to understand the exact mechanism of immune system activity and its relationship with the evolution of IBS.^13^ According to the above, it seems that psychological interventions such as mindfulness-based and compassion-focused treatments can be used as independent treatments to reduce physical and psychological symptoms and improve the performance of patients with IBS.^14^

 Mindfulness-based stress reduction (MBSR) therapy was first developed by Kabat-Zinn for a wide range of people with chronic pain and stress-related disorders.^15^ Mindfulness techniques are effective in increasing muscle relaxation and reducing worry, as well as reducing stress and anxiety.^16^ Moment-to-moment awareness of thoughts, feelings, and physical conditions can teach people to control themselves and free themselves from automatic thoughts.^17^

 Mindfulness-based stress reduction therapy reduces repetitive and negative cognitive processes, negative thinking, and memory through the activation of cognitive and emotional self-regulating mechanisms, leaving a positive effect on the final physiological, psychological, and behavioral responses.^18,19^ In fact, mindfulness as a way of life helps people to become familiar with the dual states of mind and use them consciously as an integrated mind by using meditation exercises that are integrated into daily life.^20^ The primary mechanism of mindfulness appears to be attentional self-control, as continued attention to a neutral stimulus, such as breathing, creates an appropriate attentional environment and prevents preoccupation with threatening thoughts, performance concerns, and situational appraisal.^17^

 In general, it can be said that the mindfulness-based stress reduction program is one of the new treatments that have been shown to be effective in treating most psychological and mental health problems.^21^ In addition, patients with IBS have low mindfulness. The avoidance behaviors that are related to the gastrointestinal symptoms of people with IBS are reduced by mindfulness exercises, thus reducing the symptoms in patients.^22^

 Today, the third wave of cognitive-behavioral therapy tries to create a new attitude toward human life by creating a different atmosphere. Studying the impact of new methods is certainly one way to achieve the desired psychological changes. Compassion-focused therapy is another new intervention in psychological treatments.^23^ Gilbert developed this therapy to help promote mental and emotional health by encouraging people to have compassion for themselves and others.

 The first principle of compassion-focused therapy is derived from the general systems of emotion regulation, including the threat and self-protection system, the emotion system, and the social support system. Treatment sessions consist of the connection between these systems and human thoughts and behavior.^24^ This treatment can be effective in reducing stress, anxiety, depression, and IBS.^25^ In times of suffering from severe illness and other disabilities, which are difficult to bear, compassion can be beneficial. Self-compassion is a loving and accepting position towards the undesirable aspects of oneself and one’s life, and it includes three components: self-compassion, common humanity, and mindfulness.^26^ In addition to protecting a person from negative mental states, self-compassion also plays a role in strengthening positive emotional states and thus minimizes the impact of negative experiences such as depression and anxiety.^27^

 Self-kindness refers to responding patiently and with acceptance to inadequacies, failures, and problems instead of self-criticism and self-blame. This type of response neutralizes negative emotions.^28^ Therefore, if people learn to be kind to themselves, the emotional motor system will not be easily stimulated by stress, and as a result, they will not experience severe pain and interpret stressful events in a self-supporting way. They learn not to avoid and suppress their painful feelings so they can recognize their experience in the first step and feel compassion for it.^29^ Research has shown that a large part of the therapeutic effects of IBS are due to the reduction of avoidance behaviors.^30^

 Research shows that compassion-focused and mindfulness therapy affect stress-related brain areas such as the amygdala and cingulate, resulting in a positive impact on the physiological processes and physical sensations of patients with IBS. However, the significance of the effects of these treatments on stress-related behavioral and neurobiological responses is less known.^31^

 According to the above, the necessity of conducting this research is felt. Therefore, the purpose of this research is to investigate the effectiveness of mindfulness-based stress reduction and compassion-focused on the severity of symptoms of IBS.

## Materials and Methods

 This research that was approved by the code of ethics IR.IAU.SHK.REC.1401.069 was of a semi-experimental type with a pre-test-post-test, a control group, and a 3-month follow-up. The population consisted of all people suffering from IBS in Isfahan city who visited one of the gastroenterology clinics in 2022. The sampling method was purposeful because only those who visited gastroenterology clinics were included in the research. Then, the patients were interviewed and clinically evaluated by gastroenterologists based on the Rome-IV diagnostic criteria, and 45 patients who were diagnosed with IBS and met other criteria for entering the study were randomly assigned into two experimental groups and a control group (15 in each group).

 The inclusion criteria were: receiving a definitive diagnosis of IBS by a gastroenterologist, having an age range between 20 and 40 years, having minimum education level, written consent to enter the research, absence of severe psychological diseases, absence of psychiatric medication, non-attendance at other psychoeducational programs at the same time.

 Exclusion criteria included receiving drug treatments for physical and psychological disorders during the research, absence in two consecutive sessions, and failure to perform homework. It should be noted that ethical considerations such as providing full information about the conditions of the research, confidentiality, obtaining informed consent for all participants, and using data exclusively for the purposes of the current research were fully observed in this research. In the present study, a symptom severity questionnaire and a short clinical interview were used to collect information.

###  Gastrointestinal Symptom Severity Scale

 The Gastrointestinal Symptom Severity Scale was used by gastroenterologists based on Rome 4 diagnostic criteria. The scale has a seven-point graded Likert-type from 7 to 70. Items combine into five symptom clusters: pain, defecation disorder, bloating, disease impact on daily life activities, and extra-intestinal symptoms.

 Higher scores show a higher severity of symptoms. Mazaheri and Khoshoui^32^ reported the scale’s reliability computed by using Cronbach’s alpha as 0.78. In the present study, the reliability of the questionnaire was calculated using Cronbach’s alpha coefficient on the preliminary sample as 0.82. Clinical interview: A structured clinical interview by a Ph.D. student in psychology based on DSM-5 diagnostic criteria was used to check and diagnose the absence of severe mental disorder or personality disorder and the presence of a substance abuse history.^33^

###  Procedure

 After content preparation and reviewing the background of the research, the necessary coordination was made with Isfahan Gastroenterology Clinics Association, and the necessary explanations were given regarding the objectives of the study and the type of collaboration.

 After the implementation of the questionnaires, patients with IBS who received a definitive diagnosis of the disease were invited, and after providing the necessary explanations regarding the objectives, structure, and content of the training sessions, 45 people who agreed were selected and randomly divided into two experimental groups and a control group.

 One of the experimental groups received 8 sessions of 90-minute mindfulness-based stress reduction program based on Kabat-zin’s protocol^16^ (Table 1),The other experimental group received 8 sessions of 90-minute compassion-focused therapy based on the Gilbert protocol^34^ (Table 2), while the control group did not receive any intervention during the study and only received two sessions of mindfulness-based and compassion-based combined therapy after the study termination.

**Table 1:** Treatment sessions of mindfulness-based stress reduction program Kabat-Zain^16^

**Process and therapeutic focus in sessions**	**Sessions**
Communicating and conceptualizing, explanation of disease symptoms and the need to use mindfulness training, explanation of the automatic pilot, body scan exercise, mindful eating (raisins), presenting CD No. one (body scan)	First session
Confronting obstacles, giving feedback on exercises done, doing breathing mindfulness meditation, and 10-15 minutes of sitting meditation practice.	Second session
Standing stretching exercise, mindful walking, mindful seeing and hearing, describing being in the present moment and paying attention to thoughts only as thoughts and not as facts.	Third session
Doing sitting meditation with an emphasis on the body sensations perception, deep listening to others, giving explanations regarding the judgment and the reasons for the negative judgment, homework, and presentation of CD No. 2 (mindful yoga).	Fourth session
Mindful breathing at the beginning of each session and giving feedback, describing acceptance concept and using it in dealing with problems and unpleasant experiences. Mindful sitting, body scan, reviewing of homework exercises.	Fifth session
Long-term sitting meditation, awareness of breath, sounds, and then thoughts, reviewing of homework exercises, mindful walking, standing stretching exercises, presentation of CD #3: Sitting meditation.	Sixth session
The day of silence, loving-kindness meditation training, exercises, and mindful attention to repetitive daily tasks.	Seventh session
Practicing body scan, practicing doing nothing, examining obstacles to applying techniques, reviewing past material and summarizing, preventing disease recurrence (discussing the signs of recurrence and noting important points about this), discovering potential ability in oneself, reminding, as much as you can practice.	Eights session

**Table 2:** Content of compassion-focused therapy sessions Gilbert^34^

**Process and therapeutic focus in sessions**	**Sessions**
Greetings and initial familiarization among group members, reviewing the structure of meetings, introducing the general principles and distinguishing compassion from self-pity, conceptualizing self-compassion training.	First session
Mindfulness training along with body and breathing exercises, familiarity with brain systems based on compassion, empathy training, and introducing visualization.	Second session
Describing the characteristics of compassionate people, compassion towards others, cultivating a feeling of warmth and kindness towards oneself, cultivating and understanding that others also have defects and problems (cultivating a sense of human commonalities) in contrast to the self-destructive feelings of shame, training the empty chair technique, homework assignment.	Third session
Reviewing the previous session exercise, encouraging the subjects to self-knowledge according to the learned topics and investigating their personality as a non-compassionate or compassionate person, identifying and applying exercises to building a compassionate mind, training forgiveness, and homework assignment.	Fourth session
Introduce the three-dimensional behavioral model to express the common relationship between behavior/emotions, psychological functions, and observable behavior and discuss efforts to change behavior based on it, receiving feedback and homework assignments, nurturing a compassionate mind exercise, and non-judgmental acceptance. Tolerance training.	Fifth session
Reviewing the previous session exercise, creating compassionate images, techniques, and methods of expressing compassion training (verbal, practical, and continuous compassion), integrating these practices in daily life, training the development of valuable and sublime feelings, and homework assignments.	Sixth session
Reviewing the previous session exercise, training how to write compassionate letters for oneself and others, and training how to record and keep a diary of real situations based on compassion and individual performance in that situation.	Seventh session
Training and practicing skills, reviewing and practicing the skills presented in previous sessions, training how to create a safe place, cultivating self-compassion, and finally summarizing and guidelines to integrate this method in everyday life.	Eights session

 It should be noted that all interventions were presented in person. In addition, the data related to the three phases of the research (pre-test, post-test, and 3-month follow-up) were entered into the SPSS software and finally analyzed.

## Data analysis

 To analyze the data obtained from the research tools, descriptive and inferential statistics were used. The relevant statistical analyses were performed using the statistical package of social sciences SPSS software version 25.

 Descriptive statistics indicators included frequency table and frequency percentage, mean, and standard deviation. To verify research hypotheses, analysis of variance with repeated measurements on one factor (mixed design) was used, and Bonferroni’s post hoc test was used to compare the mean scores of different groups.

 According to Table 3, the average scores of the severity of digestive symptoms of the experimental and control groups were not different from each other in the pre-test phase, but in the post-test and follow-up phases, the average scores of the severity of the symptoms of the experimental groups increased compared with the control group. The results of the Smirnov-Kolmogorov test showed that the distribution of the IBS symptom severity scores for the research variables was greater than 0.05 in the pre-test, post-test, and follow-up stages for the experimental and control groups, and the data were normal. The results of the Box’s M testrelated to the homogeneity of the covariance matrices assumption of the scores related to the severity of the IBS symptoms showed that the obtained significance level is as follows:

*P* = 0.096, F = 1.55, Box’s M = 20.87

**Table 3:** Mean and standard deviation of the experimental and control groups in relation to IBS symptom severity in the pre-test, post-test, and follow-up

**Variable**		**Stage**					
		**Pre-test**		**Post-test**		**Follow–up**	
		**Mean**	**SD**	**Mean**	**SD**	**Mean**	**SD**
Total IBS symptom severity scores	Mindfulness	37.13	6.82	24.60	5.44	23.06	5.72
	Compassion	36.86	6.25	22.46	5.23	22.46	7.89
	Control	36.46	8.95	38.53	7.75	37.60	6.69

 Therefore, it was concluded that the covariance matrices of the average scores are equal to each other (*P* = 0.001 and MW = 0.0466). Mauchly’s sphericity test statistic was: 0.001 and MW = 0.0466.

 According to Table 4, the average scores of the total severity of IBS symptoms, the measurement time (pre-test, post-test, and follow-up) (F = 51.11, *P* = 0.001), and the measurement time with the group interaction was obtained as F = 17.95, *P* = 0.001, which means that the mean scores of symptom severity were significantly different not only in different measurement times but also in different groups, which was analyzed in Table 3 using intergroup analysis. Levine’s test results to measure the equality of variances in the severity of IBS symptoms in different groups are also presented in Table 4.

**Table 4:** Results related to within-subject effects (Greenhouse-Geisser) of IBS symptom severity

**Variable **	**Source **	**Sum **	**df **	**ms**	**F **	**Sig **	**es**
Total symptom	Measurement time	2285.91	1.30	1753.85	51.11	0.001	0.549
Severity scores	Group *time	1606.35	2.60	616.23	17.95	0.001	0.461
	Error	1878.40	54.74	34.31			

 According to Table 5, the average scores of the symptoms of the experimental and control groups were significantly different from each other (*P* = 0.001, F = 13.13). The average scores of the severity of IBS symptoms differed significantly among the three groups. These differences were analyzed in Table 5 by using the Bonferroni test in pairs.

**Table 5:** The results of the intergroup effects test on the mean scores of symptom severity

**Independent variable **	**Source **	**Sum **	**df **	**ms**	**F **	**Sig **	**es **
Total symptom	Constant value	129921.06	1	129921.06	1183.11	0.001	0.966
Severity scores	Group membership	2884.13	1	1442.06	13.13	0.001	0.385
	Error	4615.13	42	109.81			

 According to the results of Table 6, there is a significant difference between the average scores of the total severity of IBS symptoms in the post-test and follow-up stages compared with the pre-test stage (*P* < 0.01); while there was no significant difference between the above average scores in the post-test and follow-up stages (*P* < 0.01). This indicates that the effectiveness of the interventions remained stable during the follow-up period, and thus the main hypothesis^2^ of the research was not confirmed.

**Table 6:** Pairwise comparison of time effect on IBS symptom severity scores of groups

**Variable**	**Pairwise comparison of research stages**		**md**	**Se**	**Sig**
Total symptom severity scores	Pretest	Posttest	8.28	1.13	0.001
		Follow up	9.11	1.19	0.001
	Posttest	follow up	0.822	0.522	0.368

## Discussion

 The purpose of the present study was to compare the effectiveness of the mindfulness-based stress reduction program and compassion-focused therapy. The research findings showed that both methods of mindfulness-based stress reduction program and compassion-focused therapy are equally effective in reducing the average severity of the IBS symptoms. This finding is in line with the results of studies by Ahmadi and colleagues,^35^ Seyyedjafari,^36^ and Akhouri and colleagues^37^ regarding the effectiveness of compassion-focused therapy, as well as with the results of Erfan Pashing et al,^38^ Naliboff et al,^39^ and Gaylord et al^40^ regarding the effectiveness of mindfulness interventions on improving the IBS symptoms.

 In explaining this finding, first of all, a clear connection between this disease and psychological and neurological factors should be noted. Psychological factors and the autonomic nervous system are related to the etiology of IBS. The relationship between digestive diseases and psychological disorders returns to the brain-gut connection, which is called the brain-gut axis. The amygdala, hippocampus, and frontal cortex areas can modulate the intestinal function and play a part in the regulation of emotions such as mood, anxiety, negative emotions, pain, and cognitive behaviors such as problem-solving, planning, searching for information, and finally, in cultivating social behaviors, coping skills and psychological well-being.^41^

 It can be stated that although these two treatments, as third-wave behavioral therapy treatments, have many commonalities,^42^ each of them was able to reduce the severity of the patients’ symptoms by their somewhat different mechanisms of action. In fact, the mindfulness-based stress reduction program made patients more aware of the severity of their symptoms through the development of stable and non-reactive awareness about internal (cognitive, emotional, and sensory) and external experiences (environmental and social).

 Attentional control is defined as the sustained focus on an object or target, for example, the self or sensation, during mindfulness practice that often involves the transference, orientation, and executive control networks of attention. Controlling attention in the initial stage of mindfulness practice is an obvious process that requires conscious effort and restraint. However, a skilled meditator is able to control his attention with less effort or by using a method that requires little effort to maintain focus in awareness.

 In addition, mindfulness practice teaches a unique focus on present moment experience. Therefore, it can relieve negative emotional experiences in a receptive and non-judgmental manner.^43^ Moreover, the expansion of stable attention and non-judgmental and non-reactive awareness can increase a wide range of different advantages such as physical relaxation, emotional balance, change in self-judgment and its improvement, self-awareness, and communication with the inner world and the capacity to accept pain and severity of symptoms in people.^43^ On the other hand, research indicates that in patients suffering from IBS, the cingulate part of the brain, which is related to attention and response selection processes, shows more activity, while the activity of this part of the brain is accompanied by the unpleasant feeling of pain.^44^

 Therefore, both mindfulness and compassion-focused interventions can positively influence the physiological processes and bodily sensations of these patients as they make it easier for the patients to accept and deal with their symptoms. Self-compassion exercises emphasize relaxation techniques, calm mind, self-compassion, and mindfulness, which will play a significant role in calming the mind and reducing stress and negative thoughts.^45,46^ Compassionate mind training is the first technique in compassion-focused therapy. Compassionate mind refers to strategies that help people experience compassion and create different aspects of it for themselves and others.^47^

 The goal is to create motivation, sympathy, sensitivity, and distress tolerance in a compassionate way through the use of special exercises. These exercises help people develop a non-judgmental and non-blaming perspective. People learn not to avoid or repress their painful feelings; thereby, they become able to recognize their experience and have compassion for it. Compassion-centered therapy does not mean avoiding pain and suffering or trying to relieve it. Compassionate therapy is not about avoiding or mitigating pain and suffering but rather a way of coping with pain and suffering.^31^

 In this approach, distinctive aspects such as an emphasis on compassion and the use of compassionate imagery have been added to various kinds of traditional cognitive-behavioral approaches, with 3 particular attention to the mindfulness of clients and therapists, and the focus is on emotion regulation model, and therapeutic interventions to create specific patterns, emotion regulation, brain states, and personal experiences are used, which underlie the change process.^31^

 Another finding is that the effectiveness of the interventions remained stable during the 3-month follow-up. This finding is consistent with the results of Hashemi and Gorji,^22^ and Pirkhaefi et al.^48^ In explaining this finding, it had been shown in previous studies that mindfulness-based treatments reduce physical symptoms in patients with IBS, whose symptoms are related to negative emotions and stress.^48^

 Mindfulness-based and compassion-focused interventions teach a person to create a new context for communication with the outer and the inner world and, in this way, create relatively stable effects.^49^ It appears that modifying the experience of stress in patients with the help of mindfulness therapy can benefit patients. Therefore, to help change patients’ symptoms, it is necessary to reduce their anxiety first, which is why mindfulness and compassion-focused therapy are so effective in improving the level and severity of patients’ symptoms because treatments based on mindfulness, including yoga techniques, reduce anxiety by utilizing mindfulness and relaxation processes and, as a result, reduce the severity of patients’ symptoms.^50^

 As mentioned earlier, avoidance behaviors are accompanied by digestive symptoms in patients. Therefore, by utilizing mechanisms such as emotion regulation, body awareness, and re-evaluation, mindfulness-based treatment can diminish individuals’ avoidance of their emotions. Consequently, this can result in a reduction of avoidance behaviors and potentially aid in symptom alleviation for patients with IBS.^51,52^

## Conclusion

 Based on the results of the present research, mindfulness-based stress reduction program and compassion-focused therapy as third-wave cognitive-behavioral treatments have a substantial impact in reducing the symptom severity of patients with IBS and these interventions could be used to treat these patients. Given that IBS has biological, neurological, and psychological dimensions, according to the results of this study, it is recommended that psychological treatments, especially mindfulness-based stress reduction program and compassion-focused therapy, be utilized as an adjunct to drug treatments to improve patients’ outcomes and also prevent the physical and psychological symptoms recurrence and reduce treatment costs.

 A limitation of the study is that the statistical population was confined to a small number of participants referring to the Shafa Parse Gastroenterology Clinic in Isfahan, which may limit the generalizability of the results. This study employed a convenience sampling method, which should be considered. According to the limitations of the current study, it is recommended that a similar study with a larger number of participants be conducted in the future.
